# Supplementary material for: Implementation outcomes of HIV self-testing in low- and middle- income countries: A scoping review
Source: PLoS One. 2021 May 3;16(5):e0250434. doi: 10.1371/journal.pone.0250434 (PMC8092786; doi:10.1371/journal.pone.0250434)
Supplement: S1 File — Word document file containing sample search strategy for Pubmed MEDLINE. (DOCX) [file pone.0250434.s001.docx]

**S1 File. Search Strategy for PubMed/Medline**

(((((((("developing country"[tiab] OR "developing countries"[tiab] OR "developing nation"[tiab] OR "developing nations"[tiab] OR "developing population"[tiab] OR "developing populations"[tiab] OR "developing world"[tiab] OR "less developed country"[tiab] OR "less developed countries"[tiab] OR "less developed nation"[tiab] OR "less developed nations"[tiab] OR "less developed world"[tiab] OR "lesser developed countries"[tiab] OR "lesser developed nations"[tiab] OR "under developed country"[tiab] OR "under developed countries"[tiab] OR "under developed nations"[tiab] OR "under developed world"[tiab] OR "underdeveloped country"[tiab] OR "underdeveloped countries"[tiab] OR "underdeveloped nations"[tiab] OR "underdeveloped population"[tiab] OR "underdeveloped world"[tiab] OR "middle income country"[tiab] OR "middle income countries"[tiab] OR "middle income nation"[tiab] OR "middle income nations"[tiab] OR "middle income population"[tiab] OR "middle income populations"[tiab] OR "low income country"[tiab] OR "low income countries"[tiab] OR "low income nation"[tiab] OR "low income nations"[tiab] OR "low income population"[tiab] OR "low income populations"[tiab] OR "lower income country"[tiab] OR "lower income countries"[tiab] OR "lower income nations"[tiab] OR "lower income population"[tiab] OR "lower income populations"[tiab] OR "underserved countries"[tiab] OR "underserved nations"[tiab] OR "underserved population"[tiab] OR "underserved populations"[tiab] OR "under served population"[tiab] OR "under served populations"[tiab] OR "deprived countries"[tiab] OR "deprived population"[tiab] OR "deprived populations"[tiab] OR "poor country"[tiab] OR "poor countries"[tiab] OR "poor nation"[tiab] OR "poor nations"[tiab] OR "poor population"[tiab] OR "poor populations"[tiab] OR "poor world"[tiab] OR "poorer countries"[tiab] OR "poorer nation"[tiab] OR "poorer nations"[tiab] OR "poorer population"[tiab] OR "poorer populations"[tiab] OR "developing economy"[tiab] OR "developing economies"[tiab] OR "less developed economy"[tiab] OR "less developed economies"[tiab] OR "underdeveloped economies"[tiab] OR "middle income economy"[tiab] OR "middle income economies"[tiab] OR "low income economy"[tiab] OR "low income economies"[tiab] OR "low gdp"[tiab] OR "low gnp"[tiab] OR "low gross domestic"[tiab] OR "low gross national"[tiab] OR "lower gdp"[tiab] OR "lower gross domestic"[tiab] OR lmic[tiab] OR lmics[tiab] OR "third world"[tiab] OR "lami country"[tiab] OR "lami countries"[tiab] OR "transitional country"[tiab] OR "transitional countries"[tiab]) OR ("developing country"[ot] OR "developing countries"[ot] OR "developing nation"[ot] OR "developing nations"[ot] OR "developing world"[ot] OR "less developed country"[ot] OR "less developed countries"[ot] OR "less developed nation"[ot] OR "under developed countries"[ot] OR "underdeveloped country"[ot] OR "underdeveloped countries"[ot] OR "middle income country"[ot] OR "middle income countries"[ot] OR "middle income nations"[ot] OR "middle income population"[ot] OR "low income country"[ot] OR "low income countries"[ot] OR "low income population"[ot] OR "low income populations"[ot] OR "lower income countries"[ot] OR "underserved population"[ot] OR "underserved populations"[ot] OR "under served population"[ot] OR "under served populations"[ot] OR "poor country"[ot] OR "poor countries"[ot] OR "poor nation"[ot] OR "poor nations"[ot] OR "poor population"[ot] OR "developing economy"[ot] OR "developing economies"[ot] OR "less developed economies"[ot] OR "low income economy"[ot] OR "low income economies"[ot] OR "low gdp"[ot] OR lmic[ot] OR lmics[ot] OR "third world"[ot] OR "lami countries"[ot] OR "transitional country"[ot] OR "transitional countries"[ot])) OR (Africa[pl] OR Afghanistan[pl] OR Albania[pl] OR Algeria[pl] OR Angola[pl] OR Argentina[pl] OR Armenia[pl] OR Azerbaijan[pl] OR Bahrain[pl] OR Bangladesh[pl] OR Barbados[pl] OR Benin[pl] OR Belarus[pl] OR Belize[pl] OR Bolivia[pl] OR Bosnia[pl] OR Herzegovina[pl] OR Botswana[pl] OR Brazil[pl] OR Bulgaria[pl] OR Burkina Faso[pl] OR Cambodia[pl] OR (Khmer[All Fields] AND Republic[pl]) OR Cameroon[pl] OR (Central[All Fields] AND ("african continental ancestry group"[MeSH Terms] OR ("african"[All Fields] AND "continental"[All Fields] AND "ancestry"[All Fields] AND "group"[All Fields]) OR "african continental ancestry group"[All Fields] OR "african"[All Fields]) AND Republic[pl]) OR Chile[pl] OR China[pl] OR Colombia[pl] OR (("comoros"[MeSH Terms] OR "comoros"[All Fields] OR "comoro"[All Fields]) AND Islands[pl]) OR Congo[pl] OR Costa Rica[pl] OR Cote d'Ivoire[pl] OR Croatia[pl] OR Cuba[pl] OR Cyprus[pl] OR Czech Republic[pl] OR Slovakia[pl] OR (Slovak[All Fields] AND Republic[pl]) OR Dominican Republic[pl] OR Ecuador[pl] OR Egypt[pl] OR (United[All Fields] AND ("arabs"[MeSH Terms] OR "arabs"[All Fields] OR "arab"[All Fields]) AND Republic[pl]) OR El Salvador[pl] OR Estonia[pl] OR Ethiopia[pl] OR Fiji[pl] OR (Gabonese[All Fields] AND Republic[pl]) OR Georgia Republic[pl] OR (Georgian[All Fields] AND Republic[pl]) OR Ghana[pl] OR Greece[pl] OR Guatemala[pl] OR Guinea[pl] OR Guam[pl] OR Guiana[pl] OR Guyana[pl] OR Haiti[pl] OR Honduras[pl] OR Hungary[pl] OR India[pl] OR Indonesia[pl] OR Iran[pl] OR Iraq[pl] OR Jamaica[pl] OR Jordan[pl] OR Kazakhstan[pl] OR Kenya[pl] OR Korea[pl] OR Kyrgyzstan[pl] OR (Kyrgyz[All Fields] AND Republic[pl]) OR Latvia[pl] OR Lebanon[pl] OR Lesotho[pl] OR Libya[pl] OR Lithuania[pl])) OR (Macedonia[pl] OR Madagascar[pl] OR (Malagasy[All Fields] AND Republic[pl]) OR Malaysia[pl] OR Malawi[pl] OR Mali[pl] OR Malta[pl] OR (Marshall[All Fields] AND Islands[pl]) OR (Agalega[All Fields] AND Islands[pl]) OR Mexico[pl] OR Moldova[pl] OR Mongolia[pl] OR Montenegro[pl] OR Morocco[pl] OR Mozambique[pl] OR Burma[pl] OR Nepal[pl] OR New Caledonia[pl] OR Nicaragua[pl] OR Nigeria[pl] OR (Northern[All Fields] AND Mariana[All Fields] AND Islands[pl]) OR Oman[pl] OR Pakistan[pl] OR Panama[pl] OR Paraguay[pl] OR Peru[pl] OR Philippines[pl] OR Poland[pl] OR Portugal[pl] OR Puerto Rico[pl] OR Romania[pl] OR Russia[pl] OR Rwanda[pl] OR Samoa[pl] OR (Samoan[All Fields] AND Islands[pl]) OR (Navigator[All Fields] AND Islands[pl]) OR Saudi Arabia[pl] OR Senegal[pl] OR Serbia[pl] OR Montenegro[pl] OR Sierra Leone[pl] OR Slovenia[pl] OR Sri Lanka[pl] OR (Solomon[All Fields] AND Islands[pl]) OR South Africa[pl] OR Sudan[pl] OR Surinam[pl] OR Syria[pl] OR Tanzania[pl] OR Thailand[pl] OR Togo[pl] OR (Togolese[All Fields] AND Republic[pl]) OR Trinidad[pl] OR Tobago[pl] OR Tunisia[pl] OR Turkey[pl] OR Turkmenistan[pl] OR Uganda[pl] OR Ukraine[pl] OR Uruguay[pl] OR Uzbekistan[pl] OR Uzbek[All Fields] OR Venezuela[pl] OR Vietnam[pl] OR Zambia[pl] OR Zimbabwe[pl] AND (Search[All Fields] AND Africa[tiab]) OR Asia[tiab] OR Caribbean[tiab] OR West Indies[tiab] OR South America[tiab] OR Latin America[tiab] OR Central America[tiab] OR Afghanistan[tiab] OR Albania[tiab] OR Algeria[tiab] OR Angola[tiab] OR Antigua[tiab] OR Barbuda[tiab] OR Argentina[tiab] OR Armenia[tiab] OR Armenian[tiab] OR Aruba[tiab] OR Azerbaijan[tiab] OR Bahrain[tiab] OR Bangladesh[tiab] OR Barbados[tiab] OR Benin[tiab] OR Byelarus[tiab] OR Byelorussian[tiab] OR Belarus[tiab] OR Belorussian[tiab] OR Belorussia[tiab] OR Belize[tiab] OR Bhutan[tiab] OR Bolivia[tiab] OR Bosnia[tiab] OR Herzegovina[tiab] OR Hercegovina[tiab] OR Botswana[tiab] OR Brasil[tiab] OR Brazil[tiab] OR Bulgaria[tiab] OR Burkina Faso[tiab] OR Burkina Fasso[tiab] OR Upper Volta[tiab] OR Burundi[tiab] OR Urundi[tiab] OR Cambodia[tiab] OR Khmer Republic[tiab] OR Kampuchea[tiab] OR Cameroon[tiab] OR Cameroons[tiab] OR Cameron[tiab] OR Cape Verde[tiab] OR Central African Republic[tiab] OR Chad[tiab] OR Chile[tiab] OR China[tiab] OR Colombia[tiab] OR Comoros[tiab] OR Comoro Islands[tiab] OR Comores[tiab] OR Mayotte[tiab] OR Congo[tiab] OR Zaire[tiab] OR Costa Rica[tiab] OR Cote d'Ivoire[tiab] OR Ivory Coast[tiab] OR Croatia[tiab] OR Cuba[tiab] OR Cyprus[tiab] OR Czechoslovakia[tiab] OR Czech Republic[tiab] OR Slovakia[tiab] OR Slovak Republic[tiab] OR Djibouti[tiab] OR French Somaliland[tiab] OR Dominica[tiab] OR Dominican Republic[tiab] OR East Timor[tiab] OR (East[All Fields] AND Timur[tiab]) OR Timor Leste[tiab] OR Ecuador[tiab] OR Egypt[tiab] OR United Arab Republic[tiab] OR El Salvador[tiab] OR Eritrea[tiab] OR Estonia[tiab] OR Ethiopia[tiab] OR Fiji[tiab] OR Gabon[tiab] OR Gabonese Republic[tiab] OR Gambia[tiab] OR Gaza[tiab] OR Georgia Republic[tiab] OR Georgian Republic[tiab] OR Ghana[tiab] OR Gold Coast[tiab] OR Greece[tiab] OR Grenada[tiab] OR Guatemala[tiab] OR Guinea[tiab] OR Guam[tiab] OR Guiana[tiab] OR Guyana[tiab] OR Haiti[tiab] OR Honduras[tiab] OR Hungary[tiab] OR India[tiab] OR Maldives[tiab] OR Indonesia[tiab] OR Iran[tiab] OR Iraq[tiab] OR Isle of Man[tiab] OR Jamaica[tiab] OR Jordan[tiab] OR Kazakhstan[tiab] OR Kazakh[tiab] OR Kenya[tiab] OR Kiribati[tiab] OR Korea[tiab] OR Kosovo[tiab] OR Kyrgyzstan[tiab] OR Kirghizia[tiab] OR Kyrgyz Republic[tiab] OR Kirghiz[tiab] OR Kirgizstan[tiab] OR "Lao PDR"[tiab] OR Laos[tiab] OR Latvia[tiab] OR Lebanon[tiab] OR Lesotho[tiab] OR Basutoland[tiab] OR Liberia[tiab] OR Libya[tiab] OR Lithuania[tiab])) OR (Macedonia[tiab] OR Madagascar[tiab] OR Malagasy Republic[tiab] OR Malaysia[tiab] OR Malaya[tiab] OR Malay[tiab] OR Sabah[tiab] OR Sarawak[tiab] OR Malawi[tiab] OR Nyasaland[tiab] OR Mali[tiab] OR Malta[tiab] OR Marshall Islands[tiab] OR Mauritania[tiab] OR Mauritius[tiab] OR Agalega Islands[tiab] OR Mexico[tiab] OR Micronesia[tiab] OR Middle East[tiab] OR Moldova[tiab] OR Moldovia[tiab] OR Moldovian[tiab] OR Mongolia[tiab] OR Montenegro[tiab] OR Morocco[tiab] OR Ifni[tiab] OR Mozambique[tiab] OR Myanmar[tiab] OR Myanma[tiab] OR Burma[tiab] OR Namibia[tiab] OR Nepal[tiab] OR Netherlands Antilles[tiab] OR New Caledonia[tiab] OR Nicaragua[tiab] OR Niger[tiab] OR Nigeria[tiab] OR Northern Mariana Islands[tiab] OR Oman[tiab] OR Muscat[tiab] OR Pakistan[tiab] OR Palau[tiab] OR Palestine[tiab] OR Panama[tiab] OR Paraguay[tiab] OR Peru[tiab] OR Philippines[tiab] OR Philipines[tiab] OR Phillipines[tiab] OR Phillippines[tiab] OR Poland[tiab] OR Portugal[tiab] OR Puerto Rico[tiab] OR Romania[tiab] OR Rumania[tiab] OR Roumania[tiab] OR Russia[tiab] OR Russian[tiab] OR Rwanda[tiab] OR Ruanda[tiab] OR Saint Kitts[tiab] OR St Kitts[tiab] OR Nevis[tiab] OR Saint Lucia[tiab] OR St Lucia[tiab] OR Saint Vincent[tiab] OR St Vincent[tiab] OR Grenadines[tiab] OR Samoa[tiab] OR Samoan Islands[tiab] OR (Navigator[All Fields] AND Island[tiab]) OR (Navigator[All Fields] AND Islands[tiab]) OR Sao Tome[tiab] OR Saudi Arabia[tiab] OR Senegal[tiab] OR Serbia[tiab] OR Montenegro[tiab] OR Seychelles[tiab] OR Sierra Leone[tiab] OR Slovenia[tiab] OR Sri Lanka[tiab] OR Ceylon[tiab] OR Solomon Islands[tiab] OR Somalia[tiab] OR Sudan[tiab] OR Suriname[tiab] OR Surinam[tiab] OR Swaziland[tiab] OR Syria[tiab] OR Tajikistan[tiab] OR Tadzhikistan[tiab] OR Tadjikistan[tiab] OR Tadzhik[tiab] OR Tanzania[tiab] OR Thailand[tiab] OR Togo[tiab] OR Togolese Republic[tiab] OR Tonga[tiab] OR Trinidad[tiab] OR Tobago[tiab] OR Tunisia[tiab] OR Turkey[tiab] OR Turkmenistan[tiab] OR Turkmen[tiab] OR Uganda[tiab] OR Ukraine[tiab] OR Uruguay[tiab] OR USSR[tiab] OR Soviet Union[tiab] OR Union of Soviet Socialist Republics[tiab] OR Uzbekistan[tiab] OR Uzbek[All Fields] OR Vanuatu[tiab] OR New Hebrides[tiab] OR Venezuela[tiab] OR Vietnam[tiab] OR Viet Nam[tiab] OR West Bank[tiab] OR Yemen[tiab] OR Yugoslavia[tiab] OR Zambia[tiab] OR Zimbabwe[tiab] OR Rhodesia[tiab])) OR (Africa[ot] OR Asia[ot] OR Caribbean[ot] OR West Indies[ot] OR South America[ot] OR Latin America[ot] OR Central America[ot] OR Afghanistan[ot] OR Albania[ot] OR Algeria[ot] OR Angola[ot] OR Antigua[ot] OR Barbuda[ot] OR Argentina[ot] OR Armenia[ot] OR Armenian[ot] OR Aruba[ot] OR Azerbaijan[ot] OR Bahrain[ot] OR Bangladesh[ot] OR Barbados[ot] OR Benin[ot] OR Belarus[ot] OR Belorussian[ot] OR Belorussia[ot] OR Belize[ot] OR Bhutan[ot] OR Bolivia[ot] OR Bosnia[ot] OR Herzegovina[ot] OR Hercegovina[ot] OR Botswana[ot] OR Brasil[ot] OR Brazil[ot] OR Bulgaria[ot] OR Burkina Faso[ot] OR (Upper[All Fields] AND Volta[ot]) OR Burundi[ot] OR Cambodia[ot] OR (Khmer[All Fields] AND Republic[ot]) OR Kampuchea[ot] OR Cameroon[ot] OR Cameron[ot] OR Cape Verde[ot] OR Central African Republic[ot] OR Chad[ot] OR Chile[ot] OR China[ot] OR Colombia[ot] OR Comoros[ot] OR Comoro Islands[ot] OR Mayotte[ot] OR Congo[ot] OR Zaire[ot] OR Costa Rica[ot] OR Cote d'Ivoire[ot] OR Ivory Coast[ot] OR Croatia[ot] OR Cuba[ot] OR Cyprus[ot] OR Czechoslovakia[ot] OR Czech Republic[ot] OR Slovakia[ot] OR Slovak Republic[ot] OR Djibouti[ot] OR (French[All Fields] AND Somaliland[ot]) OR Dominica[ot] OR Dominican Republic[ot] OR East Timor[ot] OR (East[All Fields] AND Timur[ot]) OR Timor Leste[ot] OR Ecuador[ot] OR Egypt[ot] OR (United[All Fields] AND Arab Republic[ot]) OR El Salvador[ot] OR Eritrea[ot] OR Estonia[ot] OR Ethiopia[ot] OR Fiji[ot] OR Gabon[ot] OR (Gabonese[All Fields] AND Republic[ot]) OR Gambia[ot] OR Gaza[ot] OR Ghana[ot] OR Gold Coast[ot] OR Greece[ot] OR Grenada[ot] OR Guatemala[ot] OR Guinea[ot] OR Guam[ot] OR Guiana[ot] OR Guyana[ot] OR Haiti[ot] OR Honduras[ot] OR Hungary[ot] OR India[ot] OR Maldives[ot] OR Indonesia[ot] OR Iran[ot] OR Iraq[ot] OR Isle of Man[ot] OR Jamaica[ot] OR Jordan[ot] OR Kazakhstan[ot] OR Kazakh[ot] OR Kenya[ot] OR Kiribati[ot] OR Korea[ot] OR Kosovo[ot] OR Kyrgyzstan[ot] OR Kirghizia[ot] OR Kyrgyz Republic[ot] OR "Lao PDR"[ot] OR Laos[ot] OR Latvia[ot] OR Lebanon[ot] OR Lesotho[ot] OR Liberia[ot] OR Libya[ot] OR Lithuania[ot])) OR (Macedonia[ot] OR Madagascar[ot] OR Malagasy Republic[ot] OR Malaysia[ot] OR Malaya[ot] OR Malay[ot] OR Sabah[ot] OR Sarawak[ot] OR Malawi[ot] OR Nyasaland[ot] OR Mali[ot] OR Malta[ot] OR Marshall Islands[ot] OR Mauritania[ot] OR Mauritius[ot] OR (Agalega[All Fields] AND Islands[ot]) OR Mexico[ot] OR Micronesia[ot] OR Middle East[ot] OR Moldova[ot] OR Mongolia[ot] OR Montenegro[ot] OR Morocco[ot] OR Mozambique[ot] OR Myanmar[ot] OR Burma[ot] OR Namibia[ot] OR Nepal[ot] OR Netherlands Antilles[ot] OR New Caledonia[ot] OR Nicaragua[ot] OR Niger[ot] OR Nigeria[ot] OR Northern Mariana Islands[ot] OR Oman[ot] OR Muscat[ot] OR Pakistan[ot] OR Palau[ot] OR Palestine[ot] OR Panama[ot] OR Paraguay[ot] OR Peru[ot] OR Philippines[ot] OR Philipines[ot] OR Poland[ot] OR Portugal[ot] OR Puerto Rico[ot] OR Romania[ot] OR Rumania[ot] OR Roumania[ot] OR Russia[ot] OR Russian[ot] OR Rwanda[ot] OR Ruanda[ot] OR Saint Kitts[ot] OR St Kitts[ot] OR Nevis[ot] OR Saint Lucia[ot] OR St Lucia[ot] OR Saint Vincent[ot] OR St Vincent[ot] OR Grenadines[ot] OR Samoa[ot] OR (Samoan[All Fields] AND Islands[ot]) OR (Navigator[All Fields] AND Island[ot]) OR (Navigator[All Fields] AND Islands[ot]) OR Sao Tome[ot] OR Saudi Arabia[ot] OR Senegal[ot] OR Serbia[ot] OR Montenegro[ot] OR Seychelles[ot] OR Sierra Leone[ot] OR Slovenia[ot] OR Sri Lanka[ot] OR Ceylon[ot] OR Solomon Islands[ot] OR Somalia[ot] OR Sudan[ot] OR Suriname[ot] OR Surinam[ot] OR Swaziland[ot] OR Syria[ot] OR Tajikistan[ot] OR Tadzhikistan[ot] OR Tanzania[ot] OR Thailand[ot] OR Togo[ot] OR (Togolese[All Fields] AND Republic[ot]) OR Tonga[ot] OR Trinidad[ot] OR Tobago[ot] OR Tunisia[ot] OR Turkey[ot] OR Turkmenistan[ot] OR Turkmen[ot] OR Uganda[ot] OR Ukraine[ot] OR Uruguay[ot] OR USSR[ot] OR Soviet Union[ot] OR Union of Soviet Socialist Republics[ot] OR Uzbekistan[ot] OR Uzbek[All Fields] OR Vanuatu[ot] OR (New[All Fields] AND Hebrides[ot]) OR Venezuela[ot] OR Vietnam[ot] OR Viet Nam[ot] OR West Bank[ot] OR Yemen[ot] OR Yugoslavia[ot] OR Zambia[ot] OR Zimbabwe[ot] OR Rhodesia[ot])) OR ("developing countries"[MeSH Terms:noexp] OR "africa"[MeSH Terms:noexp] OR "africa, northern"[MeSH Terms:noexp] OR "africa south of the sahara"[MeSH Terms:noexp] OR "africa, central"[MeSH Terms:noexp] OR "africa, eastern"[MeSH Terms:noexp] OR "africa, southern"[MeSH Terms:noexp] OR "africa, western"[MeSH Terms:noexp] OR "asia"[MeSH Terms:noexp] OR "asia, central"[MeSH Terms:noexp] OR "asia, southeastern"[MeSH Terms:noexp] OR "asia, western"[MeSH Terms:noexp] OR "caribbean region"[MeSH Terms:noexp] OR "west indies"[MeSH Terms:noexp] OR "south america"[MeSH Terms:noexp] OR "latin america"[MeSH Terms:noexp] OR "central america"[MeSH Terms:noexp] OR "afghanistan"[MeSH Terms:noexp] OR "albania"[MeSH Terms:noexp] OR "algeria"[MeSH Terms:noexp] OR "american samoa"[MeSH Terms:noexp] OR "angola"[MeSH Terms:noexp] OR "Antigua and Barbuda"[Mesh:noexp] OR "argentina"[MeSH Terms:noexp] OR "armenia"[MeSH Terms:noexp] OR "azerbaijan"[MeSH Terms:noexp] OR "bahrain"[MeSH Terms:noexp] OR "bangladesh"[MeSH Terms:noexp] OR "barbados"[MeSH Terms:noexp] OR "benin"[MeSH Terms:noexp] OR "republic of belarus"[MeSH Terms:noexp] OR "belize"[MeSH Terms:noexp] OR "bhutan"[MeSH Terms:noexp] OR "bolivia"[MeSH Terms:noexp] OR "bosnia and herzegovina"[MeSH Terms:noexp] OR "botswana"[MeSH Terms:noexp] OR "brazil"[MeSH Terms:noexp] OR "bulgaria"[MeSH Terms:noexp] OR "burkina faso"[MeSH Terms:noexp] OR "burundi"[MeSH Terms:noexp] OR "cambodia"[MeSH Terms:noexp] OR "cameroon"[MeSH Terms:noexp] OR "cabo verde"[MeSH Terms:noexp] OR "central african republic"[MeSH Terms:noexp] OR "chad"[MeSH Terms:noexp] OR "chile"[MeSH Terms:noexp] OR "china"[MeSH Terms:noexp] OR "colombia"[MeSH Terms:noexp] OR "comoros"[MeSH Terms:noexp] OR "congo"[MeSH Terms:noexp] OR "costa rica"[MeSH Terms:noexp] OR "cote d'ivoire"[MeSH Terms:noexp] OR "croatia"[MeSH Terms:noexp] OR "cuba"[MeSH Terms:noexp] OR "cyprus"[MeSH Terms:noexp] OR "czechoslovakia"[MeSH Terms:noexp] OR "czech republic"[MeSH Terms:noexp] OR "slovakia"[MeSH Terms:noexp] OR "djibouti"[MeSH Terms:noexp] OR "Democratic Republic of the Congo"[Mesh:noexp] OR "dominica"[MeSH Terms:noexp] OR "dominican republic"[MeSH Terms:noexp] OR "timor-leste"[MeSH Terms:noexp] OR "ecuador"[MeSH Terms:noexp] OR "egypt"[MeSH Terms:noexp] OR "el salvador"[MeSH Terms:noexp] OR "eritrea"[MeSH Terms:noexp] OR "estonia"[MeSH Terms:noexp] OR "ethiopia"[MeSH Terms:noexp] OR "fiji"[MeSH Terms:noexp] OR "gabon"[MeSH Terms:noexp] OR "gambia"[MeSH Terms:noexp] OR "Georgia (Republic)"[Mesh:noexp] OR "ghana"[MeSH Terms:noexp] OR "greece"[MeSH Terms:noexp] OR "grenada"[MeSH Terms:noexp] OR "guatemala"[MeSH Terms:noexp] OR "guinea"[MeSH Terms:noexp] OR "guinea-bissau"[MeSH Terms:noexp] OR "guam"[MeSH Terms:noexp] OR "guyana"[MeSH Terms:noexp] OR "haiti"[MeSH Terms:noexp] OR "honduras"[MeSH Terms:noexp] OR "hungary"[MeSH Terms:noexp] OR "india"[MeSH Terms:noexp] OR "indonesia"[MeSH Terms:noexp] OR "iran"[MeSH Terms:noexp] OR "iraq"[MeSH Terms:noexp] OR "jamaica"[MeSH Terms:noexp] OR "jordan"[MeSH Terms:noexp] OR "kazakhstan"[MeSH Terms:noexp] OR "kenya"[MeSH Terms:noexp] OR "korea"[MeSH Terms:noexp] OR "kosovo"[MeSH Terms:noexp] OR "kyrgyzstan"[MeSH Terms:noexp] OR "laos"[MeSH Terms:noexp] OR "latvia"[MeSH Terms:noexp] OR "lebanon"[MeSH Terms:noexp] OR "lesotho"[MeSH Terms:noexp] OR "liberia"[MeSH Terms:noexp] OR "libya"[MeSH Terms:noexp] OR "lithuania"[MeSH Terms:noexp] OR "macedonia (republic)"[MeSH Terms:noexp] OR "madagascar"[MeSH Terms:noexp] OR "malaysia"[MeSH Terms:noexp] OR "malawi"[MeSH Terms:noexp] OR "mali"[MeSH Terms:noexp] OR "malta"[MeSH Terms:noexp] OR "mauritania"[MeSH Terms:noexp] OR "mauritius"[MeSH Terms:noexp] OR "mexico"[MeSH Terms:noexp] OR "micronesia"[MeSH Terms:noexp] OR "middle east"[MeSH Terms:noexp] OR "moldova"[MeSH Terms:noexp] OR "mongolia"[MeSH Terms:noexp] OR "montenegro"[MeSH Terms:noexp] OR "morocco"[MeSH Terms:noexp] OR "mozambique"[MeSH Terms:noexp] OR "myanmar"[MeSH Terms:noexp] OR "namibia"[MeSH Terms:noexp] OR "nepal"[MeSH Terms:noexp] OR "netherlands antilles"[MeSH Terms:noexp] OR "new caledonia"[MeSH Terms:noexp] OR "nicaragua"[MeSH Terms:noexp] OR "niger"[MeSH Terms:noexp] OR "nigeria"[MeSH Terms:noexp] OR "oman"[MeSH Terms:noexp] OR "pakistan"[MeSH Terms:noexp] OR "palau"[MeSH Terms:noexp] OR "panama"[MeSH Terms:noexp] OR "papua new guinea"[MeSH Terms:noexp] OR "paraguay"[MeSH Terms:noexp] OR "peru"[MeSH Terms:noexp] OR "philippines"[MeSH Terms:noexp] OR "poland"[MeSH Terms:noexp] OR "portugal"[MeSH Terms:noexp] OR "puerto rico"[MeSH Terms:noexp] OR "romania"[MeSH Terms:noexp] OR "russia"[MeSH Terms:noexp] OR "Russia (Pre-1917)"[Mesh:noexp] OR "rwanda"[MeSH Terms:noexp] OR "Saint Kitts and Nevis"[Mesh:noexp] OR "saint lucia"[MeSH Terms:noexp] OR "Saint Vincent and the Grenadines"[Mesh:noexp] OR "samoa"[MeSH Terms:noexp] OR "saudi arabia"[MeSH Terms:noexp] OR "senegal"[MeSH Terms:noexp] OR "serbia"[MeSH Terms:noexp] OR "montenegro"[MeSH Terms:noexp] OR "seychelles"[MeSH Terms:noexp] OR "sierra leone"[MeSH Terms:noexp] OR "slovenia"[MeSH Terms:noexp] OR "sri lanka"[MeSH Terms:noexp] OR "somalia"[MeSH Terms:noexp] OR "south africa"[MeSH Terms:noexp] OR "sudan"[MeSH Terms:noexp] OR "suriname"[MeSH Terms:noexp] OR "swaziland"[MeSH Terms:noexp] OR "syria"[MeSH Terms:noexp] OR "tajikistan"[MeSH Terms:noexp] OR "tanzania"[MeSH Terms:noexp] OR "thailand"[MeSH Terms:noexp] OR "togo"[MeSH Terms:noexp] OR "tonga"[MeSH Terms:noexp] OR "Trinidad and Tobago"[Mesh:noexp] OR "tunisia"[MeSH Terms:noexp] OR "turkey"[MeSH Terms:noexp] OR "turkmenistan"[MeSH Terms:noexp] OR "uganda"[MeSH Terms:noexp] OR "ukraine"[MeSH Terms:noexp] OR "uruguay"[MeSH Terms:noexp] OR "ussr"[MeSH Terms:noexp] OR "uzbekistan"[MeSH Terms:noexp] OR "vanuatu"[MeSH Terms:noexp] OR "venezuela"[MeSH Terms:noexp] OR "vietnam"[MeSH Terms:noexp] OR "yemen"[MeSH Terms:noexp] OR "yugoslavia"[MeSH Terms:noexp] OR "zambia"[MeSH Terms:noexp] OR "zimbabwe"[MeSH Terms:noexp])) AND ((((“Self Care”[MeSH] OR “Self Administration”[MeSH]) AND ("Diagnostic Tests, Routine"[Mesh] OR test[tiab] OR tests[tiab] OR testing[tiab])) OR self-test*[tiab] OR “self test”[tiab] OR “self tests”[tiab] OR “self testing”[tiab] OR ((home[tiab] OR home-based[tiab] OR unsupervised[tiab]) AND (test[tiab] OR tests[tiab] OR testing[tiab])) OR Oraquick[tiab]) AND ("HIV"[MeSH] OR "HIV Seropositivity"[MeSH] OR "HIV Infections"[MeSH] OR HIV[tiab]))
