# Supplementary material for: Implementation outcomes of HIV self-testing in low- and middle- income countries: A scoping review
Source: PLoS One. 2021 May 3;16(5):e0250434. doi: 10.1371/journal.pone.0250434 (PMC8092786; doi:10.1371/journal.pone.0250434)
Supplement: Attachment — Submitted filename: Rivera HIVST ScR - PLOS One - response letter.docx [file pone.0250434.s004.docx]

7 January 2020

Joerg Heber, PhD

Editor-in-Chief, PLOS ONE

Dear Dr. Heber,

We previously submitted this manuscript to PLOS Medicine and PLOS ONE (PONE-D-20-29284). We appreciate the constructive feedback from the editorial team and have exerted effort to address their concerns. We feel that we have addressed them sufficiently and our manuscript should now meet PLOS ONE standards. We detailed below response to each comment we received.

Sincerely,

Adovich Rivera, MD

**DETAILED RESPONSE TO COMMENTS:**

Comment 1: We have noticed that the literature review was completed in January 2019.

To address this comment and comply with PLOS ONE standards, we have conducted a supplementary search to cover peer-reviewed literature published from January 2019 to September 2020. This update yield 100 new articles bringing the final number of articles in our review to 206. We have updated the results reflecting any new information brought by the new articles. The changes are too numerous to describe in this letter, but we have attached a marked-up version of the manuscript to highlight sections of the text that have been altered.

Comment 2: During our initial evaluation we have also noticed that no list of included study characteristics is included in the Results section.

We appreciate this comment and have included an updated Table 1 that provides an overview of study characteristics of the 206 articles (see below). We opted to use descriptive statistics instead of showing a table that provides detailed information per study due to the size of our review (n=206 articles). We agree that having structured information per study is important and provided this as supporting material (see S2 File).

Table 1. Summary Characteristics of Included Studies

|  | Overall  (n = 206) | Africa  (n = 155) | Americas  (n = 11) | Asia  (n = 40) | p-value |
| --- | --- | --- | --- | --- | --- |
| Key Population (%) | | | | | |
| MSM | 56 ( 27.2) | 11 ( 7.1) | 10 ( 90.9) | 35 ( 87.5) | <0.001 |
| FSW | 25 ( 12.1) | 21 ( 13.5) | 0 ( 0.0) | 4 ( 10.0) | 0.371 |
| HCW | 23 ( 11.2) | 20 ( 12.9) | 1 ( 9.1) | 2 ( 5.0) | 0.358 |
| Pregnant women | 19 ( 9.2) | 18 ( 11.6) | 0 ( 0.0) | 1 ( 2.5) | 0.115 |
| Transgender women | 9 ( 4.4) | 0 ( 0.0) | 2 ( 18.2) | 7 ( 17.5) | <0.001 |
| Teenagers | 46 ( 22.3) | 38 ( 24.5) | 0 ( 0.0) | 8 ( 20.0) | 0.156 |
| Type of Specimen (%) | | | | | <0.001 |
| Blood | 21 ( 10.2) | 9 ( 5.8) | 1 ( 9.1) | 11 ( 27.5) |  |
| Oral | 99 ( 48.1) | 88 ( 56.8) | 5 ( 45.5) | 6 ( 15.0) |  |
| Both | 27 ( 13.1) | 15 ( 9.7) | 2 ( 18.2) | 10 ( 25.0) |  |
| Not Specified | 59 ( 28.6) | 43 ( 27.7) | 3 ( 27.3) | 13 ( 32.5) |  |
| Type of Supervision (%) | | | | | 0.012 |
| Unsupervised | 84 ( 40.8) | 68 ( 43.9) | 4 ( 36.4) | 12 ( 30.0) |  |
| Supervised | 14 ( 6.8) | 6 ( 3.9) | 0 ( 0.0) | 8 ( 20.0) |  |
| Not Specified | 83 ( 40.3) | 60 ( 38.7) | 6 ( 54.5) | 17 ( 42.5) |  |
| Both supervision types | 25 ( 12.1) | 21 ( 13.5) | 1 ( 9.1) | 3 ( 7.5) |  |
| Type of Methods (%) | | | | | 0.155 |
| Mixed methods | 23 ( 11.2) | 21 ( 13.5) | 0 ( 0.0) | 2 ( 5.0) |  |
| Qualitative only | 54 ( 26.2) | 44 ( 28.4) | 2 ( 18.2) | 8 ( 20.0) |  |
| Quantitative only | 129 ( 62.6) | 90 ( 58.1) | 9 ( 81.8) | 30 ( 75.0) |  |
| Study Design (%) | | | | | 0.546 |
| Modelling | 3 ( 1.5) | 3 ( 1.9) | 0 ( 0.0) | 0 ( 0.0) |  |
| Observational | 149 (72.3) | 114 (73.5) | 9 (81.8) | 26 (65.0) |  |
| Interventional | 54 (26.2) | 38 (24.5) | 2 (18.2) | 14 (35.0) |  |
| a) Quasi-experimental | 32 (20.3) | 18 (16.2) | 2 (18.2) | 12 (33.3) |  |
| b) Randomized trial | 22 (13.9) | 20 (18.0) | 0 ( 0.0) | 2 ( 5.6) |  |
| Implementation Outcome (%) | | | | | |
| Acceptability | 122 (59.2) | 93 (60.0) | 10 (90.9) | 19 (47.5) | 0.032 |
| Adoption | 103 (50.0) | 73 (47.1) | 4 (36.4) | 26 (65.0) | 0.085 |
| Appropriateness | 56 (27.2) | 46 (29.7) | 1 ( 9.1) | 9 (22.5) | 0.253 |
| Cost | 16 ( 7.8) | 15 ( 9.7) | 0 ( 0.0) | 1 ( 2.5) | 0.195 |
| Feasibility | 24 (11.7) | 20 (12.9) | 1 ( 9.1) | 3 ( 7.5) | 0.614 |
| Fidelity | 23 (11.2) | 17 (11.0) | 1 ( 9.1) | 5 (12.5) | 0.939 |
| Penetration | 2 ( 1.0) | 2 ( 1.3) | 0 ( 0.0) | 0 ( 0.0) | 0.717 |
| Sustainment | 1 ( 0.5) | 1 ( 0.6) | 0 ( 0.0) | 0 ( 0.0) | 0.848 |
| Linkage to Care | 59 (28.6) | 38 (24.5) | 4 (36.4) | 17 (42.5) | 0.068 |
| Safety | 40 (19.4) | 32 (20.6) | 1 ( 9.1) | 7 (17.5) | 0.609 |

Notes: MSM – males who have sex with males, FSW – female sex worker, HCW – healthcare worker
