# Supplementary material for: Implementation outcomes of HIV self-testing in low- and middle- income countries: A scoping review
Source: PLoS One. 2021 May 3;16(5):e0250434. doi: 10.1371/journal.pone.0250434 (PMC8092786; doi:10.1371/journal.pone.0250434)
Supplement: Attachment — Submitted filename: HIVST - response to comments on version 2.docx [file pone.0250434.s005.docx]

**DETAILED RESPONSE TO REVIEW COMMENTS:**

**Comment 1:** introduction seems too succinct? expanding further on what is already known about the topic and how your research fills the gap might be helpful.

Response: We agree with this comment and have expanded our review. We added more context about implementation research as well as added more description of the findings of previous reviews. We also revised our description of what our review adds to the field.

Changes in text (page 3 to 4, lines 28 to 65):

“It is recognized as a key tool to help achieve the global 90-90-90 targets, specifically, ensuring that 90% of people with HIV are aware of their status [3,4]. Many countries are in the process of integrating HIV-ST into their national HIV/AIDS programs [5]. This process can easily become complicated and poor implementation can lead to countries not gaining any benefits (e.g., address the HIV testing gap) from the new technology. Fortunately, implementation science has emerged in response to this problem of introducing new technology to health systems and health organizations have recognized that implementation itself requires a scientific approach and reviews of the current evidence are valuable in this approach.

One approach to organizing this evidence base is according to implementation outcomes. Using Proctor et al.’s framework [6], implementation outcomes are different from the usual outcomes studied in clinical or health services research. Usually, outcomes in health research are patient outcomes (e.g., satisfaction or improvement in health and function) or quality of care outcomes (e.g., efficiency, safety, and timeliness) which are end products of an intervention. Meanwhile, implementation outcomes are indicators of success that are “the effects of deliberate and purposive actions to implement new treatments, practices, and services.” Further, Proctor et al. proposes that only programs with successful implementation outcomes will achieve maximal effectiveness and quality. These eight outcomes are *acceptability, adoption, appropriateness, costs, feasibility, fidelity, penetration,* and *sustainability*. By identifying the relevant conditions necessary to achieve HIV-ST implementation outcomes, we can better understand how best to implement this technology for a given setting.

Another important factor in implementation is the context (i.e., where HIV-ST will be implemented). For example, the design of the national health system puts constraints on how HIV ST can be delivered to the end-user. The dominant modes of HIV transmission in the country are contextual factors that influences what populations will be prioritized by countries. Finally, the level of stigma faced by at-risk populations affects how they would react to HIV-ST. While there have been several reviews on HIV-ST implementation outcomes, these reviews primarily included studies conducted in high-income countries [7–11] with a couple of reviews focused on implementation in Africa [12,13]. We agree that the reviews are very much useful. They found evidence that HIV-ST was acceptable to a wide variety of patients and providers and warn about issues that may hinder successful implementation like the high cost of the kit, inadequate linkage to care, and the complexity of packet instructions. But there is a huge diversity of low- and middle-income countries (LMIC) contexts, findings and lessons for upper-income countries or from Africa may not always be applicable to other LMIC settings. Our review is important to guide individual country efforts in planning implementation research and programs of HIV-ST in their local settings.”

**Comment 2:** The authors provided too many kinds of implementation outcomes. Some kinds of implementation could be integrated, such as Adoption and penetration. Or pls give clearer definition on these types. Currently, it's not easy to differentiate the difference.

**Comment 3:** Page 5, line 92-95. These sentences were awkward. Suggesting to rephrase them.

Response: We are thankful for these comments. In order to be consistent with Proctor’s framework and facilitate application of our findings to other implementation research, we decided to keep the original implementation outcomes. We do recognize that some outcomes (e.g. adoption and penetration, described in lines 92-95) sound similar, so we updated the operational definitions we used in the review.

Changes in Text (page 5 to 6, lines 90 to 117):

“We followed Proctor et al.’s definition of implementation outcomes [6] for this study with some modifications to facilitate categorization of studies (See S2 File for notes on definitions). During extraction, we recognized how of *safety* and *linkage to care* are intricately linked to successful implementation of HIV-ST, so we opted to include them in our review as additional outcomes. We did not expand the search for these two outcomes, rather, we focused on extracting only data on safety and linkage if a paper also reports one of the eight implementation outcomes.

One pair of outcomes that needed to be delineated operationally are *feasibility* and *fidelity*. Both outcomes relate to how well actual implementation aligns with the ideal implementation. We differentiate the two by separating the technology (HIV-ST) from the implementation program (i.e., how HIV-ST is reaching the end-user). We then treat *feasibility* as those related to successful use of HIV-ST as a piece of technology by the end-user. As such we included error rates and real-world diagnostic performance under feasibility. For *fidelity*, we focused on compliance of stakeholders to implementation strategies or described standard procedures employed within the HIV-ST program. Examples of these would be the procedure of giving two kits to each potential user or performing a follow-up call after self-testing.

Another pair that we had to differentiate was *adoption* from *penetration*. Both are related to the degree of uptake of technology in a population and both can be measured by determining how many among the eligible are using HIV-ST but occurs at different phases of implementation. Implementation can be viewed as a process with pre-implementation, early, middle, and late phases which can be described roughly as exploration, pilot testing, early roll out, and full institutionalization of new technologies [15]. We then interpret *adoption* studies to measure uptake in the early to middle phases as well as investigate if specific strategies lead to increased uptake by the target population. Meanwhile, we viewed *penetration* as a late-stage measure that requires the technology to be available for a longer period than in *adoption* and is quantified both service access and spread. Proctor’s definition also has an added dimension of how well implementation strategies or processes have become part of usual care. Using this heuristic, we considered studies that looked at how many people have used HIV-ST before or during early implementation programs as under *adoption* and studies that measured uptake at later phases under *penetration*.”

**Comment 4:** line 357 - I may have missed this - but how did you pool your results - more detail in methods please.

Response: We appreciate this request and have added some more details about the pooling in the methods.

Changes in the text (page 7, lines 140 to 143):

“We pooled diagnostic accuracy (sensitivity and specificity) results using bivariate modeling that was implemented through the mada package [18]. Sensitivity or specificity was extracted from the text or calculated from two-by-two tables. For calculations, we excluded indeterminate test results.”

**Comment 5:** Results section (in general) could benefit from better use of subheadings. I got lost after a while.

Response: We are thankful for this suggestion. We have added subheadings to the results section to help the reader.

**Comment 6:** line 120 - are these the absolute numbers of papers found or are they percentages?

Response: We apologize for the confusion. We revised the text to include both the number of papers and the percentage in the text.

Changes in the text (page 8, lines163 to 166):

“Most of the studies were on implementation in African contexts (70%) and were situated in four countries: Kenya (33, 16%), South Africa (33, 16%), Malawi (28, 14%), and China (27, 13%) (Fig 2).”

**Comment 7:** line 142 - can you expand on why the willingness to use was consistently high among FSW, and also provide further information as to why there was marked variations for MSM?

**Comment 8:** line 147-148 - similar to above, why was there such a marked variation from 32% to 97%? highlighting some key lessons here would be helpful

**Comment 9:** Page 8, line 147. "Willingness to pay". Is there variation on this across different key populations?

Response: We thank the reviewers for these requests. We added more details about studies with lower rates and variation in willingness to pay. We think that the variation is due to how the samples were defined in the different studies. Some studies included only those who had prior HIV testing experience while others used a mixed sample (ever HIV testers and never testers). The studies with low rates included those who had never tested for HIV and might have led to the lower willingness.

Changes in Text:

Page 10, lines 187 to 194:

“In contrast, rates in MSM were more varied, ranging from 51 [73] to 99% [77] (Fig 3). We noted that the two studies (conducted in Kenya [129] and in Brazil [73]) that reported low willingness to use rates included individuals who have never tested for HIV (self-test or usual testing). This inclusion may have led to the lower measure of overall willingness. One study in South Africa [48] found that the odds of being willing to use HIV-ST was two times higher among ever testers compared to never testers. Although, we also found a study in Tanzania [86] that did not find significant differences in the willingness to use rates of ever and never testers.”

Page 11, lines 200 to 207:

“Most studies showed willingness greater than 50% across key populations. Some of the low rates are from populations with limited income such as students in Tanzania and Central African Republic [30,75] and FSW primarily working in poor neighborhoods in Central African Republic [30]. The remaining study with low WTP [79] is on MSM in China where free HIV testing is available. Their sample also included a lot of people who have never tested which may affect WTP and the way they asked the WTP question (“Willing to purchase an oral self-test kit (in the next 6 months)?”) seemed to capture both willingness to try and pay (Fig 3).”

**Comment 10:** line 184 - why was this the exception - what made this study different from the rest?

Response: We think that it is due to the comparison group used for this study. Other studies just compared it to announcements or standard practice. This study compared distribution community health workers to pharmacies as distributors in rural settings. We added this detail in the text.

Changes in the text (page 13, lines 254 to 259):

“The non-significant results in the Zimbabwe study [67] might be due to the comparison group. The Zimbabwe study had three factors in the intervention (price of kit, type of kit distributor, and promotional messaging). They found that needing to pay even just $0.50 significantly reduced uptake. In rural sites, they compared community health workers to retail stores as distributors and did not find any significant difference in uptake. They also found that type of messaging did not significantly affect uptake.”

**Comment 11:** line 203 (and elsewhere) - this paragraph is an example whereby having consistent formatting when referring to various papers would be more informative to the reader - as a minimum, you should state the setting (country) for each study so the reader understands the context of these statements.

Response: We appreciate this feedback. We took the reviewers suggestion and added the country where the study was conducted for studies we chose to highlight. We also added the key population being studied in some statements to provide more context.

**Comment 12:** Page 15, line 290. Pls check this saying. According to my field experience, this approach is acceptable.

Response: We apologize for the confusion. We agree that this approach is acceptable, and we even found evidence that this strategy is effective for distributing HIV-ST. We revised the statement to make our point clearer and avoid confusion.

Changes in the text (page 18, lines 371 to 374):

“Partner distribution is an often-used strategy for distributing HIV-ST kits. Several papers explored issues of acceptability and appropriateness [94,95,99–102,105,107,114,118,119,121,122,127,183,184,186,189,191,196] and all except one [121] were done in African countries.”

**Comment 13:** line 311 - this paragraph touches on an important topic of the potential for social harms. It would be helpful to expand this further with data from a few key studies including what was being measured, how it was done, and what did they find

Response: We agree with this reviewer and have update two sections of our results to add more details about social harms. We added details of qualitative studies in the Appropriateness section as well as more quantitative details about these events in the Safety section.

Changes in the text:

Page 19, lines 380 to 387

“Relatedly, the safety of female distributors is an important consideration in program design. Female distributors were believed to be at risk of physical violence, abandonment, and relationship dissolution. A qualitative study in Malawi [95] found that “women feared being branded as unfaithful and a possible domestic violence” if they bring up doing HIVST together. A similar study in Uganda also documented that women fear their partner’s reaction the test although, in this study, none of the participants reported serious adverse events after offering the kit to male partners except for angry reactions among those who lied about the purpose of the test [102]. Results of quantitative studies on safety and social herms are reported in the safety section below.”

Page 29, lines 602 to 605

“The occurrence of adverse events like coercive testing, self-harm after positive test results, and interpersonal violence seemed to be low across the studies that included these metrics [25,47,78,85,96,97,100,125,140,145,147,150,156,162,165,169,176,178,180,181,183,188,190,192]. Some studies reported no serious adverse advents.[78,127,144,152]”

Page 29, lines 613 to 621

“There were also other forms of harm reported in studies. In a pilot trial in Uganda [85], two of 19 distributors reported facing hostility from potential users. Marriage dissolution was raised by participants in a qualitative study in Malawi [95] but the report of the trial where the qualitative study was nested did not report occurrence of this event.[78] In a Zambian trial [145], there were 13 reported social harm events among 13,267 participants in the HIV-ST arm and these events included two cases of couple separation, three cases of emotional distress, and one cases of threat of suicide. In trial in Uganda [146], there were only two reported events among 632 HIV-ST users involving one case of verbal abuse and one case of emotional distress from a false positive result.”

**Comment 14:** line 403/404 - this sentence is awkward and has some typos in it

Response: We are thankful for pointing out this sentence. We agree and have revised our description of these studies on cost to improve clarity.

Changes in the text (page 24 to 25, lines 507 to 513):

“The design and policies of the larger HIV program where HIV-ST is integrated also affect cost-effectiveness. For example, Maheswaran et al. [221] found that HIV-ST as a complementary service of standard testing can be cost-effective from a public provider perspective if positive individuals are managed following the 2015 WHO anti-retroviral treatment guidelines but not if the 2010 guidelines were used. Meanwhile, Johnson [222] showed that adding HIV-ST to offering a home-based HIV testing program in South Africa is cost-saving, but pairing HIV-ST with antenatal care partner testing programs is only cost-effective.”

**Comment 15:** line 441 - please add citation.

Response: Upon reviewing this line, we noted some inaccuracy of our description of HIV-ST approval in China. We revised the sentence to be more accurate and added citations.

Changes in the text (page 26, lines 539 to 542):

“We see in China (one of the countries where HIV-ST is widely available through e-commerce sites [223]), that the uptake rate of ever using HIV-ST can be at <20% [175] if measured outside of HIV-ST implementation programs.”

**Comment 19:** Page 23, line 475. It was self-reported data, or confirmed with proof like photos.

Response: We reviewed the included data and found that studies were mixed in terms of how they measured receiving confirmatory testing. Some relied on self-reports without verification while others reviewed clinic records to validate self-reports. We added these details in the text.

Changes in the text (pages 27 to 28, lines 576 to 579):

“There was a mix of approaches to measuring confirmatory testing. Most studies verified receipt of testing by either having all HIVST users undergo confirmatory testing as part of the protocol or by looking at clinic records with only a third (12/36, 33%) relying solely on report by study participants.”

**Comment 20:** line 478 - what was the reason?
**Comment 21:** line 480-1 - why so low? (i.e. <20% linkage)

Response: We were unable to find concrete answers to the low linkage rates. However, we added details about these studies to provide more context and may partially explain why these rates were low.

Changes in the text (page 28, lines 589 to 597):

“Similarly, most studies that reported linkage to care of confirmed positive cases found more than 50% linkage rates [23,29,57,74,77,78,84,133,144,151,152,207] with only two reporting <20% linkage rates [81,156]. One study with low rates [81] was the pilot study in Indonesia described above. The other was a trial of HIVST distribution via female partners in Uganda that reported low linkage of males who tested positive.[156] Most studies also reported 50% or higher rates ART initiation [24,25,29,61,84,133,140,151,152,160,162,207,224–226]; only two studies (in China [168] and in South Africa [174]), both of which were focused on assessing strategies to improve uptake among males, reported <30% initiation rates. Both studies had systems in place to provide counseling and follow-up to those who tested positive but still observed low rates.”

**Comment 22:** line 509 - can you expand on the comparison between your findings and the other reviews conducted in high-income countries. What was common? different?

Response: We agree with this comment and have expanded our discussion section. We added more details about findings of previous reviews and noted similarities in our review. We also noted findings from new literature that the prior reviews were not able to capture.

Changes in the text (page 30 to 31, lines 632 to 650):

“Our key findings are aligned with findings of previous reviews that covered high income settings or have been limited to Africa [7,10–13]. Prior reviews concluded that HIV-ST is generally acceptable especially if available at a low cost and that can be used by target populations with good accuracy and low error rates. Like our review, prior reviews also found that HIV-ST was viewed as a convenient alternative to facility-testing that improves testing confidentiality, reduces stigma, and supports autonomy. These reviews also pointed out counseling and linkage as important issues that need to be tackled in implementation. The reviews generally had little information on effectiveness of adoption strategies, linkage to care, and safety and our review found several studies that tacked these issues. Notably, we found promising evidence for partner or peer-based distribution strategies. We also found quantitative studies that found issues related to linkage and safety occur but are uncommon. Despite the low rates of harm or losing to follow-up, it is important for HIV-ST programs to include features to prevent or address these issues including a surveillance and quick response system to mitigate serious events like suicide. The concept of safety should not be limited to physical harm, rather, it should include emotional or economic harm such as coercive testing, distress from positive results, couple separation, or threats of economic abandonment. Finally, while findings of our review and the prior reviews generally converge regardless of geographic region, we observed that published work was still absent in several countries. We stress the need for local research to inform each country’s policies and programs.”

**Comment 23:** consider discussing the potential loss of benefits from contact with a health professional if only using self-testing, e.g. counselling, access to testing for other STIs, vaccinations, etc...

Response: We highly appreciate this point and have integrated this in the text.

Changes in the text (page 32, lines 676 to 681):

“As HIV-ST programs are slowly integrated into existing HIV programs, the potential impact of reduced face-to-face encounters with HCW should be considered. These encounters can serve as means to deliver health promotion messages that may not be related to HIV but are still important to health. Losing these encounters may lead to unintended health consequences that may need to be considered especially in cost-effectiveness analyses using the societal perspective.”

**Comment 24:** consider using the term men who have sex with men instead

Response: We agree with this and applied this in the text. (line 166, page 8)

**Comment 25:** Page 18, line 363. Typo error. should be "nine".

**Comment 26:** line 209 - typo "social media"

**Comment 27:** line 228 - delete "ok but need to clean up"

**Comment 28:** line 412 - brackets around 2019

**Comment 29:** line 429 - there is some sloppy use of punctuation and spaces. This is but one example. Please carefully proofread your manuscript.

Response: We apologize for the typographical and grammatical errors in our submission. We reviewed the text several times to remove these errors.
